# pH Switchable Water Dispersed Photocatalytic Nanoparticles

**DOI:** 10.1002/chem.202200118

**Published:** 2022-05-12

**Authors:** Moreno Guernelli, Arianna Menichetti, Gloria Guidetti, Paolo Emidio Costantini, Matteo Calvaresi, Alberto Danielli, Raffaello Mazzaro, Vittorio Morandi, Marco Montalti

**Affiliations:** ^1^ Department of Chemistry “Giacomo Ciamician” University of Bologna Via Selmi 2 40126 Bologna Italy; ^2^ Consiglio Nazionale delle Ricerche Istituto per la Microelettronica e i Microsistemi Bologna 40129 Italy; ^3^ Department of Pharmacy and Biotechnology University of Bologna Via Selmi 3 40126 Bologna Italy

**Keywords:** fluorescence, nanoparticles, photocatalysis, reactive oxygen species (ROS), stimuli responsive materials

## Abstract

Photogeneration of Reactive Oxygen Species (ROS) finds applications in fields as different as nanomedicine, art preservation, air and water depollution and surface decontamination. Here we present photocatalytic nanoparticles (NP) that are active only at acidic pH while they do not show relevant ROS photo‐generation at neutral pH. This dual responsivity (to light and pH) is achieved by stabilizing the surface of TiO_2_ NP with a specific organic shell during the synthesis and it is peculiar of the achieved core shell‐structure, as demonstrated by comparison with commercial photocatalytic TiO_2_ NP. For the investigation of the photocatalytic activity, we developed two methods that allow real time detection of the process preventing any kind of artifact arising from post‐treatments and delayed analysis. The reversibility of the pH response was also demonstrated as well as the selective photo‐killing of cancer cells at acidic pH.

## Graphical abstract

**Photogeneration of Reactive Oxygen Species (ROS)** can be controlled in space and in time by changing the irradiation intensity and focusing the activation light. Nevertheless, action of ROS is non‐specific and the possibility of exploiting a local chemical signal as an additional control represents a great advancement. Tailored modification of the structure yields dual‐stimuli responsive nanoparticles which are photo‐active and kill cancer cells at acidic pH but not at neutral pH.

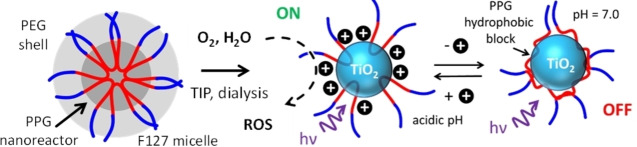

Controlled generation of Reactive Oxygen Species (ROS) upon light stimulation is a powerful strategy to enable local and selective on‐demand production of highly redox‐active species in selected environment.^[1]^ This technology finds applications that vary from cell targeted disruption in cancer therapy^[2]^ to cultural heritage preservation^[3]^ to treatment of polluted air and water.[^4^, ^5^] Light activation also findsapplication in organic synthesis.^[6]^ Recently photocatalysis‐induced ROS generation has also been proposed for inactivating COVID‐19 virus.^[7]^

ROS photo‐generation is typically achieved upon absorption of light by proper chemical species, the photocatalysts, which are molecules, aggregates, or nanoparticles (NP) that, after excitation, undergo energy‐ or electron‐ transfer processes that involve water and oxygen molecules.[^1^, ^8^] Hence, ROS generation can be switched ON/OFF in time simply by controlling the irradiation intensity, and in space by focusing the excitation light on the desired target volume. In this framework, the additional possibility of triggering the response to light by an additional chemical stimulus (local pH) promises to guarantee enhanced control of the process as well as specificity.^[9]^

Here we describe dual stimuli‐responsive nanoparticles (**DSR NP**) that efficiently generate ROS only upon simultaneous luminous and chemical stimulation. In particular these NP are poorly efficient ROS photo‐generators at neutral pH, but they produce ROS very efficiently at acidic pH. Considered the importance of local pH in some pathologies related to cancer^[10]^ and in important global processes, such as for example ocean acidification,^[11]^ the possibility of triggering ROS photo‐generation with pH promises to find application in fields as different as biology, medicine and environmental sciences.

For example, generation of ROS has already been proven to be effective in tumor microenvironment by means of Fenton reaction triggered by iron‐ and copper‐based nanoparticles.^[12]^

Semiconductor NP, such as TiO_2_ NP, have been widely used for ROS photo‐generation in the form of powder, film or solid dispersion but, very rarely, as stable water dispersed NP mostly for the difficulty of achieving a transparent suspension of monodispersed, small, photoactive NP.^[13]^ **DSR NP** we describe here i) are synthesized accordingly to a highly environmentally friendly templated method (no high temperature or pressures or solvent other than water are needed), ii) form extremely transparent water dispersions stable for months, iii) show a very high photoactivity at acidic pH but not at neutral pH.

**DSR NP** were synthesized as schematized in Scheme 1 using highly biocompatible Pluronic F 127 micelles as templating agents to control the growth of TiO_2_ formed from the hydrolysis and condensation of the molecular precursor titanium (IV) isopropoxide (TIP).^[14]^ The reaction was carried on in mild condition (50 °C during stirring) and the resulting **DSR NP** were purified by dialysis. High resolution transmission electron microscopy (HR‐TEM) images of the NP are shown in Figure 1(a). The FFT of the HR image shows the typical pattern of [1,0,0] Anatase TiO_2_ demonstrating that the **DSR NP** are crystalline and display the typical crystal lattice of Anatase phase.^[15]^ Despite a slight aggregation of the NP, probably resulting from the sample deposition, HR‐TEM image analysis allowed also to measure the size distribution of the NP and to determine the average size of the TiO_2_ core that was *d*=6.0±2.1 nm. The hydrodynamic diameter of the NP at different pH was measured by Dynamic Light scattering (DLS), results are reported in Table 1. Table 1 shows that the size of the **DSR NP** is only slightly affected by the pH in the 1.0 to 5.0 range being ∼30 nm and hence larger than the size of the TiO_2_ cores. This difference has been also reported for analogous SiO_2_ NP and attributed to the contribution of the surfactant shell in expanding the hydrodynamic diameter of the NP.^[14b]^

**Scheme 1:**
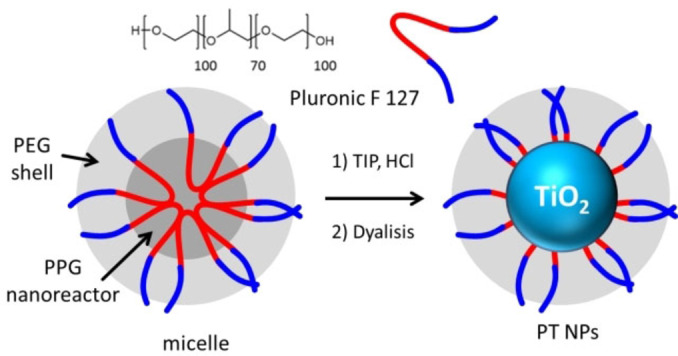
Templated synthesis of **DSR NP** by hydrolysis and condensation of the molecular precursor TIP in the PPG core of the Pluronic F127 micelles.

**Figure 1:**
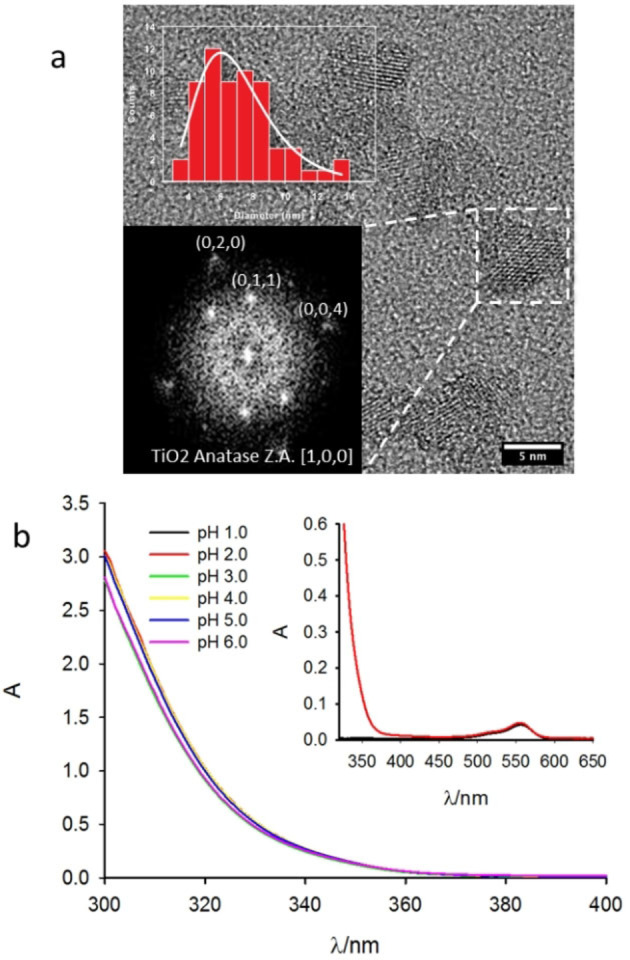
a) HR TEM images of the **DSR NP**. In the inset: top, size distribution histogram of the **DSR NP**; bottom, Fast Fourier Transform of the HR‐TEM micrograph. b) Absorption spectra of the **DSR NP** (0.2 mg/mL) at different pH. In the inset: absorption spectra of RhB (0.5 μM) in the presence (red line) and in the absence (black line) of **DSR NP**.

**Table 1:** Properties of **DSR NP** (0.2 mg/mL) as a function of pH.

pH	*d*_H_ [nm]	PDI	*ζ*‐potential [mV]	*E*_g_ [eV]	A_340nm_	% Ads	*k* ×10^3^ s^−1^	*ϕ* ×10^3^
1.0	27.1	0.23	60±7	3.3	0.277	∼0	77.4	26.7
2.0	26.4	0.24	54±6	3.3	0.277	∼0	28.4	14.4
3.0	36.0	0.29	38±9	3.3	0.245	20	16.2	2.0
4.0	33.0	0.24	33±3	3.4	0.277	32	10.6	1.8
5.0	31.7	0.20	31±5	3.4	0.270	33	1.9	0.9
6.0	55.3	0.29	18±7	3.4	0.255	20	1.4	0.5
7.0	55.0	0.27	20±4	–	–	–	1.7	–

Regarding the stability of **DSR NP** against aggregation, *ζ* potential measurements reported in Table 1 indicate large positive value at low pH, as expected for the protonation of the Ti−OH groups on the NP surface. A decrease of *ζ* potential was observed upon pH increase as expected because of the decrease of surface charge.[^15^, ^16^] Nevertheless, NP were still stable against aggregation up to pH=6.0. This stability was confirmed by the absorption spectra of the **DSR NP** suspension in the 1.0–6.0 pH range (*c*=0.2 mg/mL) shown in Figure 1(b) that clearly demonstrate the lack of any turbidity. The absorption spectra were processed to calculate the optical band‐gaps reported in Table 1. Thanks to the transparency the DSR NP suspensions could be treated like molecular solutions and their photochemical activity investigated as for homogenous systems.^[17]^

We would like to underline that this is not typically possible for largely aggregated TiO_2_ NP. In particular, thanks to the transparency it was possible to follow in real time the photocatalytic degradation of a molecular target rhodamine B (RhB) which is degraded by the photo‐generated ROS (see Figure 2), by fluorescence spectroscopy. In our setup, excitation at 340 nm was exploited both for exciting the photocatalytic **DSR NP** and RhB in solution and the fluorescence of the organic dye was detected at 590 nm. By comparing the absorption of RhB (0.5 μM) either in the presence or in the absence of **DSR NP** (0.2 mg/mL) we could conclude that only a very minor fraction (<1 %) of the excitation light is absorbed by RhB, while most of the light is absorbed by the **DSR NP**. In a typical experiment, as shown in Figure 2(b), a gradual decrease of the fluorescence of RhB was observed during irradiation of the **DSR NP**‐RhB solution because of the photodegradation of RhB.

**Figure 2:**
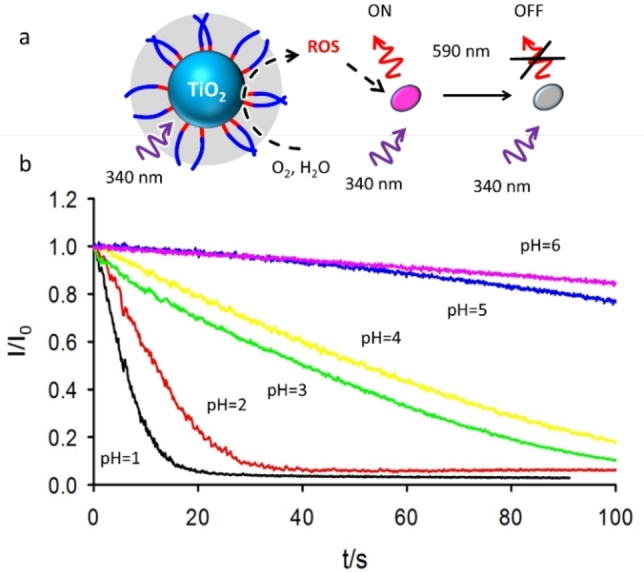
a) Scheme of the method for investigating the kinetic of photodegradation. The same beam (340 nm) is used for excitation of the photocatalyst (**DSR NP**) and of the target (RhB). Photogenerated ROS degrade RhB causing a decrease of the fluorescence. Fluorescence at 590 nm is followed to detect the concentration of RhB. b) Fluorescence at 590 nm as a function of time during irradiation at different pH (1.0–6.0).

In order to investigate the effect of pH on the process, the experiment was repeated at different pH. Since no changes in the absorption spectrum could be detected at the excitation wavelength during the experiment (and absorbance at the detection wavelength was less than 0.1) the fluorescence intensity, shown in Figure 2(b), could be directly correlated to the RhB concentration during time.^[21]^ Figure 2(b) clearly shows that photodegradation is much faster at more acidic pH being completed in about 20 s at pH=1.0 while at pH 6.0 after 100 s only 10 % of RhB molecule is degraded. In order to better compare the photodegradation rates, we fitted the first 10 s of the decays acquired at different pH with a pseudo zero‐order model according to Equation 1:

(1) $$\frac{I\left(t\right)}{I_{0}}=1-kt$$

The kinetic rate constants resulting from the fitting are shown in Table 1 and they demonstrate that photodegradation at pH=1.0 is more than 50 times faster than at pH=6.0. From a different point of view, we can observe that in the same time interval of 20 s the degradation is complete at pH=1.0 while at pH=6.0 it is almost negligible (2 %–3 %). The photodegradation experiment was also performed at pH=7.0. Although flocculation of the NP at this pH starts to be detectable, inducing partial turbidity, RhB degradation trace could be still analyzed to give the rate constant shown in Table 1. All these results demonstrate that the high photocatalytic activity of **DSR NP** can be switched ON/OFF simply by changing the pH.

In order to better understand the photodegradation mechanism, we performed a detailed photophysical analysis of the **DSR NP**‐RhB system at different pH in order to investigate the interaction between RhB and the NP. This analysis is discussed in detail in the Supporting Information. In summary, steady state fluorescence demonstrated that a minor fraction of RhB fluorescence is quenched by the **DSR NP** before irradiation in a pH dependent manner. On the other hand, time correlated single photon counting (TCSPC), and fluorescence anisotropy (FA) measurements clearly demonstrated that the detected fluorescence was just due to RhB molecules free in solution and not interacting with the **DSR NP**.

These results allowed us to conclude that a minor fraction of RhB, calculated in Table 1, is adsorbed on the **DSR NP** and its fluorescence is completely quenched. This adsorbed fraction is pH dependent since only the zwitterionic form of RhB is electrostatically adsorbed onto the positively charged DSR NP while the cationic one is not. In particular at pH 1.0 and 2.0 no adsorption is observed as expected considering that for RhB p*K*_a_ is 3.7 (see Supporting Information). These observations give important insight in the mechanism of photodegradation. Indeed, considering that at pH 1.0 and 2.0, despite no RhB molecules are adsorbed onto the **DSR NP**, the degradation is much faster than at higher pH we can conclude that degradation does not involve direct interaction between **DSR NP** and RhB, but it is mediated by the photo‐generation of ROS suggesting the mechanism schematized in Figure 2(a) that was confirmed by the following experiments. The formation of ⋅OH radicals during **DSR NP** irradiation, as a result of water oxidation, was indeed demonstrated using Coumarin as a specific probe (Figure S7), as previously proposed by Zhang and Nosaka.^[18]^ Additionally, a considerably increase of the hydroxyl radical generation rate upon acidification was observed (Figure S8, Table S1) while photodegradation of RhB upon irradiation of **DSR NP** in the absence of oxygen was not observed even at pH=3.0 as expected on the bases of the mechanism proposed in Ref. [22].

Reversibility is a relevant feature for the design of multi‐responsive nano‐devices, hence we investigated the actual possibility of switching ON/OFF the photo‐degradation activity of **DSR NP** by changing pH. To dothis we started from a suspension of **DSR NP** at pH 2.0 containing RhB 0.5 μM that was irradiated for 60 s at 340 nm. RhB degradation during this first cycle was followed by detecting the fluorescence at 590 nm shown in Figure 3(a) as a red line. The pseudo‐zero order kinetic constant was also calculated and plotted in Figure 3(b). After 60 s pH was raised to 5.0 by addition of NaOH and the RhB added to restore the 0.5 μM concentration. The fluorescence recorded during irradiation at 340 nm in this 60 s second cycle is reported as blue line in Figure 3(a), the pseudo‐zero order kinetic constant was also plotted in Figure 3(b). After 60 s irradiation the pH was lowered to 2.0 by addition of HCl and irradiation repeated for 60 s, simultaneously detecting the fluorescence as shown in Figure 3.

**Figure 3:**
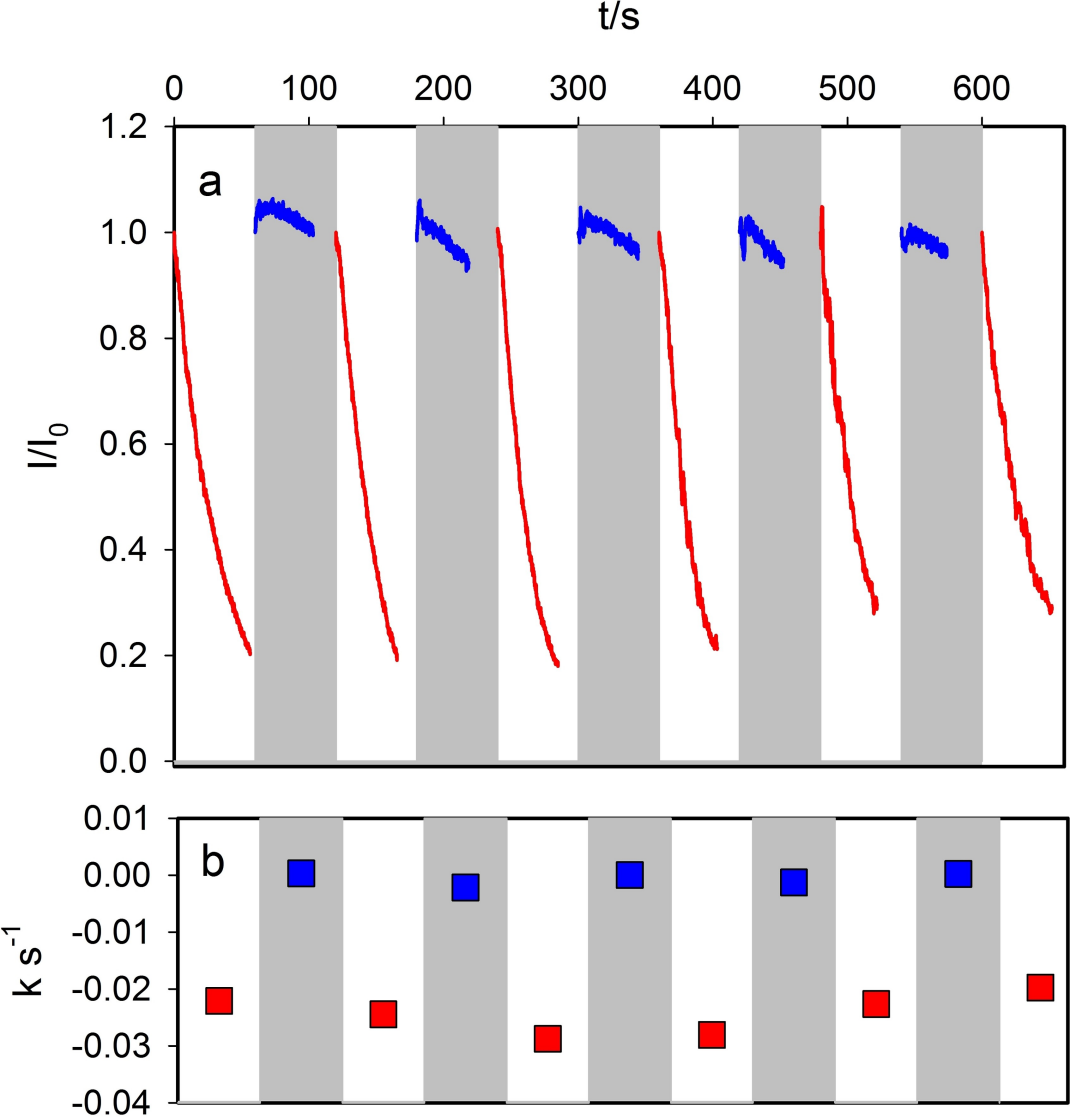
a) Cycles of photo‐catalytic degradation of RhB by **DSR NP**. Red curves show fluorescence at 590 nm at pH=2.0 and blue curves at pH=5.0. b) Pseudo‐zero order rate constant calculated for each cycle.

The experiment was continued by changing the pH alternatively from 2.0 to 5.0 by following within each cycle the RhB degradation. The fluorescence changes for eleven cycles are plotted in Figure 3 together with the rate constant calculated within each cycle (in red for cycles at pH=2.0 and blue for pH=5.0). Experimental results clearly demonstrate that photodegradation is very efficient in the cycles at pH=2.0 but it is almost negligible in the cycles at pH=5.0. In conclusion the photocatalytic activity of **DSR NP** can be reversibly switched ON‐OFF by switching the pH from 2.0 to 5.0. Considered this outstanding behavior, we wanted to demonstrate it was characteristic of our **DSR NP**, hence we compared the photocatalytic properties of **DSR NP** to the ones of a reference TiO_2_ NP sample P25.^[18]^

The photodegradation analysis based on fluorescence detection we reported is very useful to understand in detail the interaction between the target RhB and the **DSR NP** and to detect the process in real time. As a limitation it cannot be applied to turbid samples as P25 dispersions. Conventional methods, mostly based on UV‐Vis absorption detection, on the other hand typically require processing the sample after irradiation and they are not suitable for real time detection. Hence, we developed a very convenient method based on real time optical imaging of multiple samples de‐coloration during irradiation. The method is described in detail in the Supporting Information. Briefly, selected wells of a 24‐well plate were partially filled with the photocatalyst (**DSR NP** or P25 at the same concentration) suspensions containing RhB 20 μM at a known pH. The wells were than irradiated with a solar light simulating lamp and they were imaged by time‐lapsed acquisition with a color RGB CMOS camera. The fraction of light absorbed by the RhB in each well, as a function of time, was calculated by processing the images with the software package Image J. The calculated absorbance, proportional to the RhB concentration, was plotted as a function of time and fitted with a pseudo‐zero order model as shown in the Supporting Information. The effect of pH on the photocatalytic activity of the commercial P25 was very modest and only a 3.7 times decrease of the rate constant was observed going from pH 1.0 to pH 7.0 (*k*=4.4×10^−3^ s^−1^ and *k*=1.2×10^−3^ s^−1^ respectively). On the contrary for **DSR NP** the photocatalytic activity was the same of P25 at pH 1 but decreased about 50 times at pH 7 (*k*=4.7×10^−3^ s^−1^ and *k*=0.1×10^−3^ s^−1^ respectively). Hence, we could conclude that the presence of the surfactant shell induces a strong decrease of the photocatalytic activity at neutral pH. This behavior results from the interaction of the hydrophobic PPG block of the surfactant Pluronic F127 with the TiO_2_ surface.^[19]^ As schematized in Figure 4, indeed, at acidic pH the titanol groups on NPs surface undergo protonation, (p*K*_a1_=5.0, p*K*_a2_=7.8) forming a positively charged surface.^[20]^ As demonstrated by the Z‐potentials reported in Table 1 a significant decrease of the positive charge is observed at pH 6.0 and 7.0 suggesting that, in these conditions, electrostatic stabilization is only partial and steric stabilization has to be considered. The effect of Pluronic F127 surface adsorption is hence the reason of the different pH dependent ability of **DSR NP** to degrade RhB with respect to commercial P25. In fact, the experimental results demonstrate that, as schematized in Figure 4, the decreased surface polarity causes an enforcement of the hydrophobic interaction of the less polar surface with the hydrophobic PPG block section of the Pluronic F127 surfactant shielding the photoactive surface from water molecules and hence decreasing its reactivity. Hence, while Cu and Fe based materials undergo pH dependent valence variations,^[21]^ in the case of **DSR NP**, pH responsivity is due to surface protonation. With respect toother system that exploit X‐ray^[21d]^ irradiation, light‐based activation is surely less invasive but also less penetrating in living tissues.

**Figure 4:**
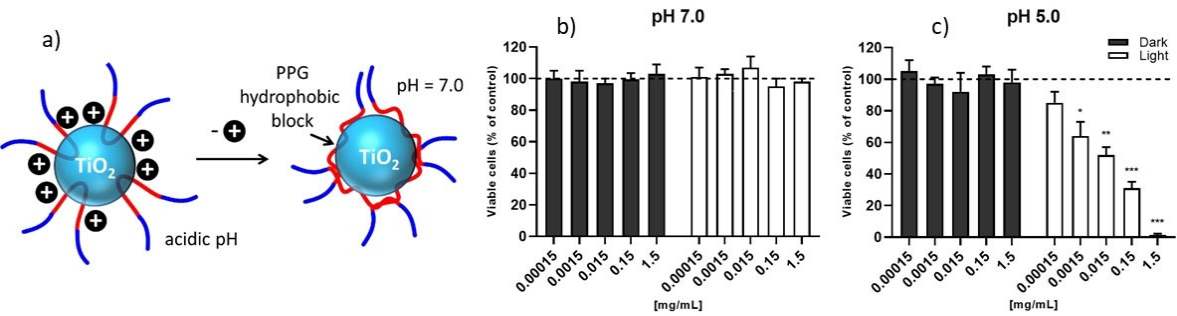
a) Schematic representation of the mechanism responsible for the decreased photocatalytic activity of **DSR NP** at pH 7.0 with respect to acidic pH. The killing of cancer cell line by DSR NP upon photo and chemical stimulation was evaluated on HeLA cell treated with DSR NP diluted from 1 : 10 (d10) to 1 : 100 000 (d100 000) in either b) PBS pH 7 or c) PBS pH 5. After treatment cells were irradiated with white lamp (white bars) or kept under dark conditions (dark grey bars). Cell viability is expressed in percentage with respect to the untreated sample (dashed line). *= p‐value <0.05, ***= p‐value <0.01, ***= p‐value <0.001.

The dual responsivity of **DSR NP** was further proven by incubating tumor cells with **DSR NP** diluted in neutral (pH 7.0) or acid (pH 5.0) buffer and by irradiating with luminous source. As a result, a significant decrease in cell viability was observed only on cells that were treated with **DSR NP** at acid pH and irradiated with light (Figure 4). Moreover, cell viability of irradiated cells is inversely proportional to **DSR NP** concentration, demonstrating the perfect dose‐dependent killing effect. Conversely, the single stimulation of **DSR NP** with either light source or acid pH did not induce any cytotoxic effect on tumor cells, proving the dual responsivity of **DSR NP**. On the contrary, as discussed in the Supporting Information, no pH dependent cancer cell photo‐killing was observed for bare commercial TiO_2_ NP (P25).

In conclusion we demonstrated that the templated synthesis of **DSR NP** show very efficient ROS photo‐generation at acidic pH while they are inactive at neutral pH. This behavior is peculiar of these NP, it cannot be observed in commercial TiO_2_ NP like P25, and it has been demonstrated to be reversible. Considered the importance of local pH in some pathologies related to cancer and in important global processes, such as for example ocean acidification, the possibility of triggering ROS photo‐generation with pH promises to find application in fields as different as biology, medicine and environmental sciences.

## Conflict of interest

The authors declare no conflict of interest.

## Supporting information

As a service to our authors and readers, this journal provides supporting information supplied by the authors. Such materials are peer reviewed and may be re‐organized for online delivery, but are not copy‐edited or typeset. Technical support issues arising from supporting information (other than missing files) should be addressed to the authors.

Supporting Information

## Data Availability

The data that support the findings of this study are available in the supplementary material of this article.
