# Supplementary material for: pH Switchable Water Dispersed Photocatalytic Nanoparticles
Source: Chemistry. 2022 May 12;28(34):e202200118. doi: 10.1002/chem.202200118 (PMC9321822; doi:10.1002/chem.202200118)
Supplement: Supplementary file 1 — Supporting Information [file CHEM-28-0-s001.pdf]

Chemistry–A European Journal

Supporting Information

pH Switchable Water Dispersed Photocatalytic Nanoparticles

Moreno Guernelli, Arianna Menichetti, Gloria Guidetti, Paolo Emidio Costantini, Matteo Calvaresi, Alberto Danielli, Raffaello Mazzaro, Vittorio Morandi, and Marco Montalti*

Materials and Methods

Reagents solvents and materials

Titanium Isopropoxide (TIP, Sigma Aldrich,>98%), Hydrochloric acid (HCl, Sigma Aldrich, 37%), Sodium Hydroxide (NaOH, Sigma Aldrich), Pluronic F127 (F127, Sigma Aldrich), Rhodamine B (RhB, Merck).

All reagents, solvents and chemicals were purchased from Sigma-Aldrich and used without modification, unless otherwise stated. Ultrapure water was produced with a Millipore system.

Synthesis of DSR NP

For the synthesis of the photocatalytic NP a water solution of the surfactant Pluronic F127 was prepared (16mM) in HCl 70 mM. Temperature was risen to 50°C and the solution was let to stabilize for 30 min. [1,2] At this point, TIP was added rapidly under stirring (1100 rpm) in order to obtain 15 mg/ml NP solution and a white precipitate was observed. Then, solution was kept at 50°C under stirring (600rpm) for 24 h. A whitish clear solution was obtained.

In the end, the solution was purified via dialysis (excluded MW 10-12 kDa) against a water solution of HCl 1 mM for 3 days.

Photophysical and photochemical measurements

UV-Vis Spectroscopy

The experiments were carried out in air-equilibrated solutions at 25 °C. UV–Vis absorption spectra were recorded with a Perkin-Elmer Lambda 650 or Perkin-Elmer Lambda 45 spectrophotometer using quartz cells with path length of 1.0 cm.

Fluorescence Spectroscopy

The fluorescence spectra were recorded with a Horiba Jobin Yvon Fluoromax-4 or a Perkin-Elmer LS-55 or an Edinburgh FLS920 equipped with a photomultiplier Hamamatsu R928 phototube. The same instrument connected to a TCC900 card was used for Time Correlated Single Photon Counting (TCSPC) experiments with an LDH-P-C 405 pulsed diode laser. The fluorescence quantum yields (uncertainty, ± 15%) according to the standard method. [3] The emission intensities were corrected taking into consideration the inner filter effect. [3] For the fluorescence anisotropy measurements an Edinburgh FLS920 equipped with Glan-Thompson polarizers was used. The data were corrected for polarization errors using the G-factor.

Dynamic Light Scattering (DLS)

DLS measurements were performed with Zetasizer Nano ZS Malvern Panalytical using PMMA semi-micro cuvettes (BRAND).

Photophysical investigation of the interaction of RhB with DSR NP at different pH

The interaction of RhB 0.5 µM with DSR NP was investigated at different pH by UV-Vis absorption, steady state and time resolved fluorescence spectroscopy and fluorescence anisotropy. In particular it is known that RhB at pH 7.0 is present in solution in the zwitterionic form with the carboxylic group which is deprotonated while it undergoes protonation forming $RhBH^+$ according to the equilibrium shown in figure S1 (pK_a=3.7). [4]

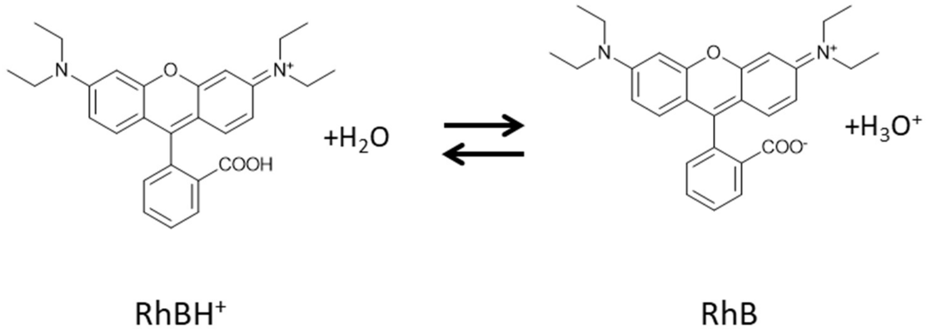

$$K_a = \frac{[\mathrm{RhB}][H_3O^+]}{[\mathrm{RhBH}^+]}$$

Figure S1. *Equilibrium of protonation of RhB.*

Because of the different polarity and charge of RhB and $RhBH^+$, these two forms are expected to interact differently with the NP surfaces.

Determination of the fraction of RhB adsorbed on DSR NP.

In order to investigate the interaction of RhB with **DSR NP** we measured the excited state lifetime of the rhodamine dye at different pH in the presence and in the absence of the NP. The resulting average excited state lifetimes are plotted in figure S2. In particular the excited state decays were acquired by Time Correlated Single Photon Counting (TCSPC) and fitted with a bi-exponential model:

$$I(t) = B_1 e^{-\frac{t}{\tau_1}} + B_2 e^{-\frac{t}{\tau_2}}$$

And the average lifetime was calculated as:

$$< \tau > = \frac{B_1\tau_1 + B_2\tau_2}{B_1 + B_2}$$

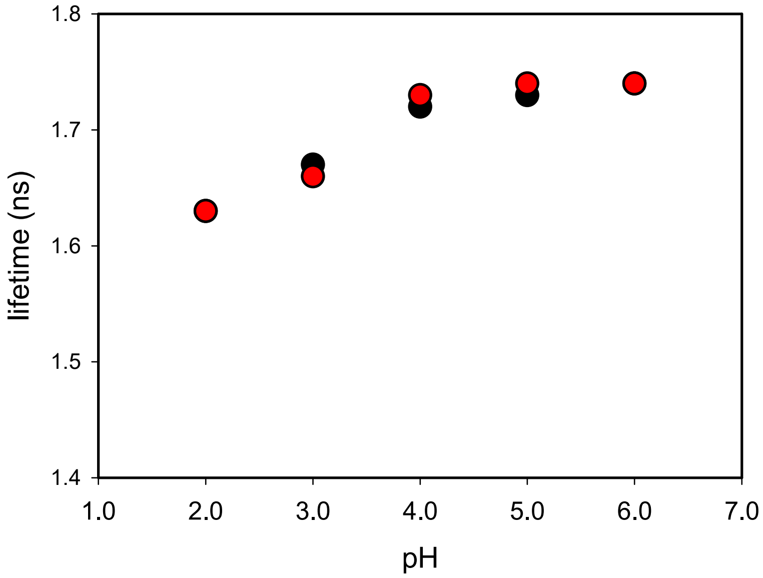

Figure S2. *Average excited state lifetime of RhB, as a function of pH, in the absence (black dots) and in the presence (red dots) of* ***DSR NP****.*

The results demonstrate that: i) the average excited state lifetime of RhB is pH independent at pH>4.0 and it decreases slightly at lower pH as expected for the formation of $RhBH^+$; ii) the behavior is identical in the presence and in the absence of **DSR NP**. These results allow to conclude that there is no effect of the presence of the NP on the equilibrium of protonation of RhB.

Fluorescence anisotropy measurements

Fluorescence anisotropy measurements allow to investigate processes of reorientation, and in particular rotation, of fluorophores occurring in the time interval between the excitation and the

photon emission. In particular in the case of the presence of RhB molecules adsorbed onto the surface of the NP rotation would become very slow and high fluorescence anisotropy values would be expected. Indeed fluorescence anisotropy measurements (λ_{exc}=530 nm) revealed fluorescence anisotropy values at λ_{exc} 600 nm (RhB fluorescence) very close to 0 (r~0) independently on the pH and on the presence or absence of **DSR NP**. This result demonstrates that all the fluorescent RhB are indeed not adsorbed on the NP surfaces.

Fluorescence quenching measurements

Considered the results of lifetime and fluorescence anisotropy experiments it is possible to rule out the presence of RhB molecules adsorbed on the NP and still fluorescent. Hence possible RhB molecules adsorbed on the NP can be only non fluorescent and hence quenched by the interaction with the NP. In this case the measured intensity or better the average fluorescence quantum yield is:

$$< \Phi >= \chi_1 \Phi_1 + \chi_2 \Phi_2 + \cdots$$

Hence if χ_1 represent the molar fraction of adsorbed and quenched rhodamine (hence Φ_1=0)

$$\chi_1 = \frac{< \Phi > - < \Phi_{NP} >}{< \Phi >}$$

Were $< \Phi >$ and $< \Phi_{NP} >$ are the average fluorescence quantum yield of rhodamine in the absence and in the presence of the NP respectively. The fluorescence intensity shown in figure S3 have been corrected in order to be proportional to the fluorescence quantum yield. It is clear that a partial, pH dependent, quenching of the fluorescence is observed. These data were used to calculate the fraction of rhodamine adsorbed onto the NP at the different pH as reported in the main text in table 1.

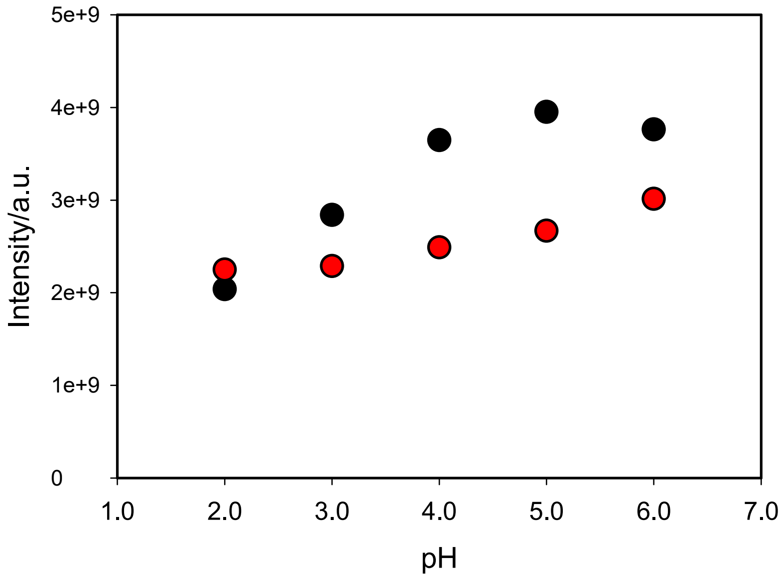

Figure S3. *Average fluorescence intensity of RhB, as a function of pH, in the absence (black dots) and in the presence (red dots) of* ***DSR NP****.*

Calculation of the optical band gap from TAUC plots

Tauc plot method is used to determine the optical bandgap in semiconductors. Tauc equation can be expressed as:

$$\alpha h\nu = A(h\nu - E_g)^n$$

Where hv is incident photon energy, Eg optical bandgap, A band tailing parameter, n transition power factor and α absorption coefficient. α is defined by Lambert's law as $\alpha(\nu)=2.303\cdot A(\nu)\cdot d^{-1}$, where A and *d* are solution absorbance and thickness, respectively [5]. The value of n denotes the transition nature:

- n=0.5 for *direct allow transitions*.
- n=1.5 for *direct forbidden transitions*.

- n=2 for *indirect allowed transitions*.
- n=3 for *indirect forbidden transitions*.

Tauc plot shows in abscissa hv (eV) and on the ordinate the quantity $(\alpha h\nu)^{1/n}$. Resulting plot shows a linear region from which it is possible to extrapolate Eg via least-squared technique, see figure S4.

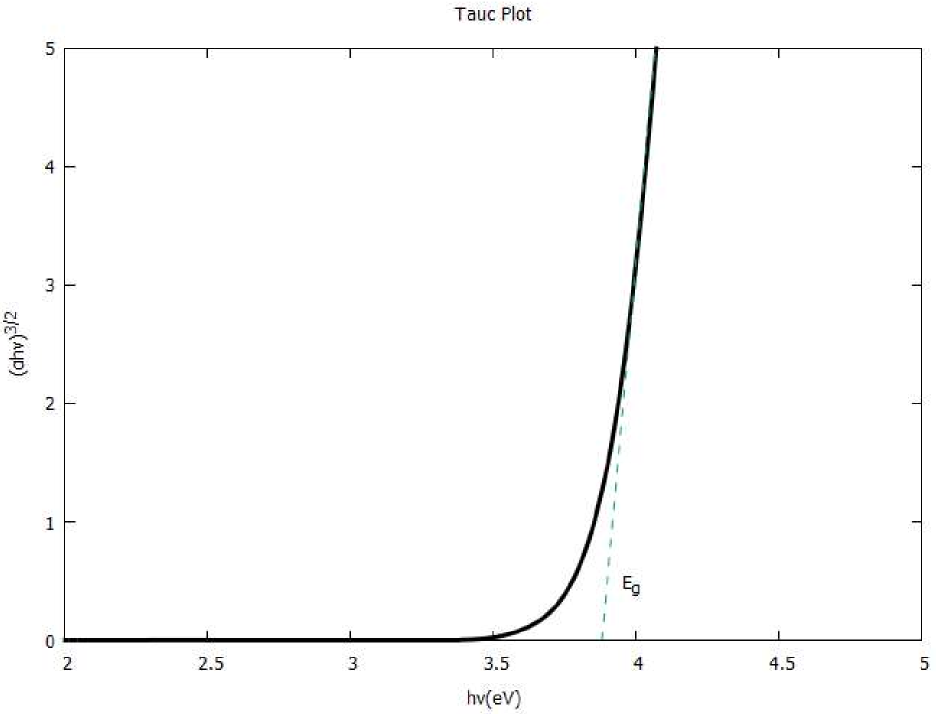

Figure S4. *Example of the method used for calculating the optical band gap.*

Analysis of RhB photodegradation by fluorescence real-time detection

From the photochemical point of view the quantum efficiency or quantum yield of a photoreaction can be defined as the number of molecules produced divided by the number of photons adsorbed by the system, hence by the PC, in the case of a photocatalyzed process.[6]

Hence this definition can be applied only for transparent samples for which the absorbance can be measured.

For kinetic investigation 6 µL of RhB (0.02mM) and 42 µL of **DSR NP** (final concentration 0.2 mg/ml) was dissolved in 3 ml of milliQ water. Solution pH was adjusted by HCl or NaOH water solutions. Resulting solution was kept under stirring (900rpm) during measurements. Irradiation (340 nm) is performed using the excitation beam of a conventional spectrofluorometer (HORIBA FluoroMax, intensity $1.1197 \cdot 10^{16}$ photon/s) and the fluorescence of RhB is detected at 580 nm in a L configuration.

Definition of photocatalytic activity and determination of quantum yield

Quantum yield was obtained dividing the total number of RhB degraded molecules, by the number of absorbed photons by **DSR NP** per unit of time. Total number of RhB molecules (#RhB) in solution could be easily calculated:

$$\#RhB = [RhB] \cdot V \cdot N_a = 1 \cdot 10^{-6} \cdot 3 \cdot 10^{-3} \cdot 6{,}022 \cdot 10^{23} \approx 18 \cdot 10^{14}$$

With [RhB] initial molar concentration of RhB, V the solution volume and Na the Avogadro's number.

The flux of absorbed photons (q_{abs}) can be obtained by:

$$Q_{abs} = Q_{in} \cdot (1 - 10^{-A}) = 1.1197 \cdot 10^{16} (1 - 10^{-0.277}) \approx 0.528 \cdot 10^{16} \text{ Ein/s}$$

With Q_{in} flux of incident photons and A PC absorbance.

Finally, quantum yields for every pH values are obtained simply by:

$$\varphi = \frac{\#\text{RhB}}{2\, Q_{abs}\, t_{1/2}}$$

Actinometers

The used actinometer is potassium ferrioxalate.

$$Fe(C_2O_4)_3{}^{3-} \xrightarrow{h\nu} Fe^{2+} + C_2O_4{}^{\cdot -} + 2C_2O_4{}^{2-}$$

$$Fe(C_2O_4)_3{}^{3-} + C_2O_4{}^{\cdot -} \rightarrow Fe^{2+} + 2CO_2 + 3C_2O_4{}^{2-}$$

The potassium ferrioxalate solution is irradiated and then Phenanthroline is added, in order to measure the absorbance of the Fe^{2+}-Phenanthroline complex at 510 nm.

Three 3,5 mL-cuvettes are prepared with 3 mL of the actinometer solution. Then, two cuvettes are irradiated by the lamp for a determined time interval while the other one is kept in dark. The absorbance at 510 nm (Fe^{2+}-Phen complex) is measured of the three samples and the difference if absorbance between the irradiated sample and the not irradiated one (ΔA(510)) is calculated.

Actinometer – Fluoromax

**Lamp**	**Xenon**
**Irradiation wavelength**	**340 nm**
**Irradiation time**	**30 s**
**Slit**	**20 nm**

The incident flux is calculated by the formula:

$$q_{p,in} = \frac{\Delta A(510)}{l \cdot \varepsilon(510)} \cdot V \cdot \frac{N_A}{\Phi \cdot t}$$

This formula is valid if the incident light is completely absorbed by the solution (A>3).

$\Delta A(510)$ = 2,1

l= 1 cm

$\varepsilon(510)$= 11100 L mol^{-1}cm^{-1}

V = volume of the irradiated solution (3 mL)

$\Phi(334 \text{ nm})$ = quantum yield at 334 nm = 1,23

$$q_{p,in} = \frac{2,1}{1\,cm \cdot 11100\, L\, mol^{-1} cm^{-1}} \cdot 3 \cdot\ 10^{-3} L \cdot \frac{6,02 \cdot 10^{23} mol^{-1}}{1,23 \cdot 30} = 0,9 \cdot 10^{16}$$

Rhodamine B degradation analysis by time lapsed color imaging.

To compare the photocatalytic activity of **DSR NP** and P25, a 24 well-plate was filled with a solution of RhB 0.02 mM. PBS 1x buffer (pH 7) and a water solution of HCl 0.1 M (pH 1) were used as solvents to further compare the photocatalytic behavior in neutral and acidic environment. At this point, PC (0.2mg/ml) were added. P25 was previously dispersed in water by sonication (30 min). Then, the plate was irradiated by a solar simulator lamp for 30 min (OSRAM, 25cm).

For image acquisition CMOS camera (Imaging Source DFK23UP031, sensor MT9P031, 2592x1944 pixels). The R channel of the RGB sensor is sensitive to wavelength λ>550 nm and hence is suitable to measure the component of light adsorbed by rhodamine B. The residual fraction of RhB, hence the concentration of RhB (c) with respect to the initial one (c0) was determined by measuring IR/I where IR is the intensity measured on the red channel and I=IR+IG+IB is the total intensity as the sum of the red, green (IG) and blue (IB):

$$c(t)/c_0 = \frac{I_R(t)}{I_T(t)} \frac{I_T^0}{I_R^0}$$

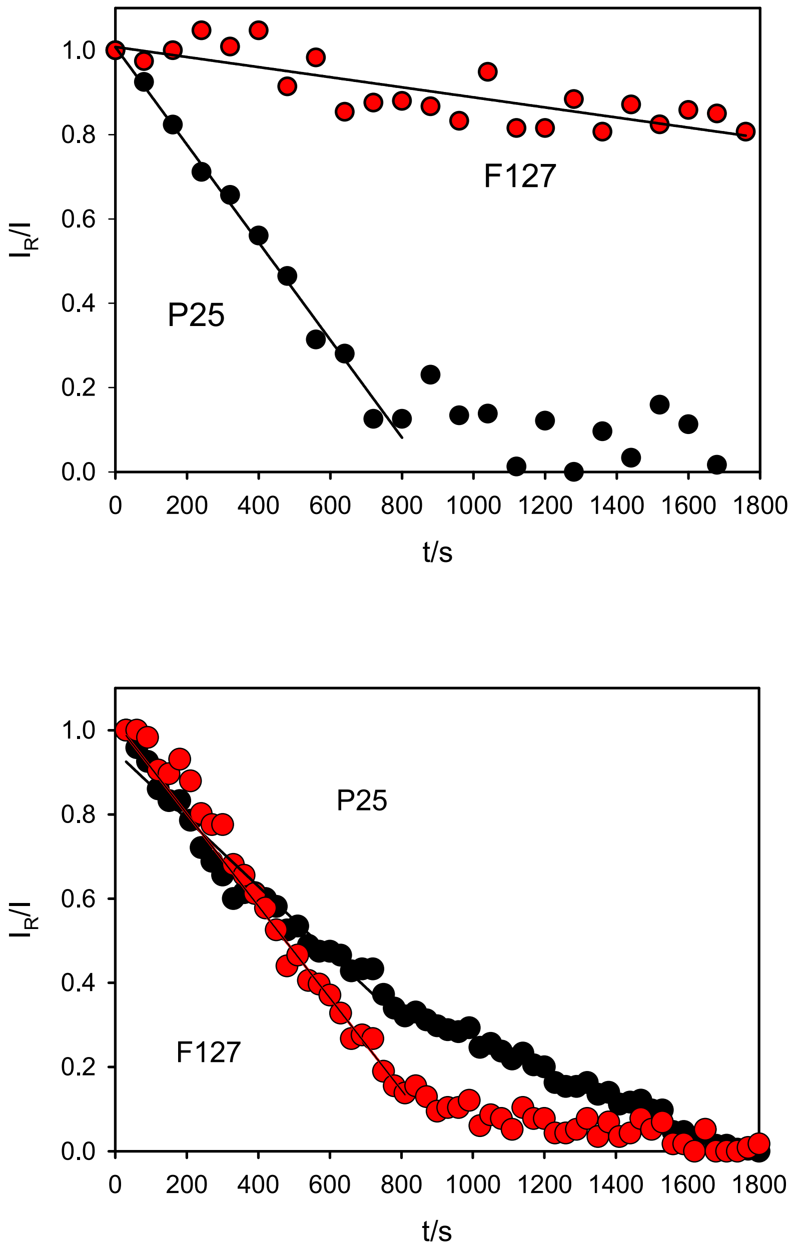

Figure S5. *Elaboration of photodegradation kinetics. Top) Comparison of photoactivity at pH 1 between* ***DSR NP*** *(red dots) and P25 (black dots): signals proportional to the concentration of RhB are plotted as a function of the irradiation time. Bottom) Comparison of photoactivity at pH 7 between* ***DSR NP*** *(red dots) and P25 (black dots): signals proportional to the concentration of RhB are plotted as a function of the irradiation time.*

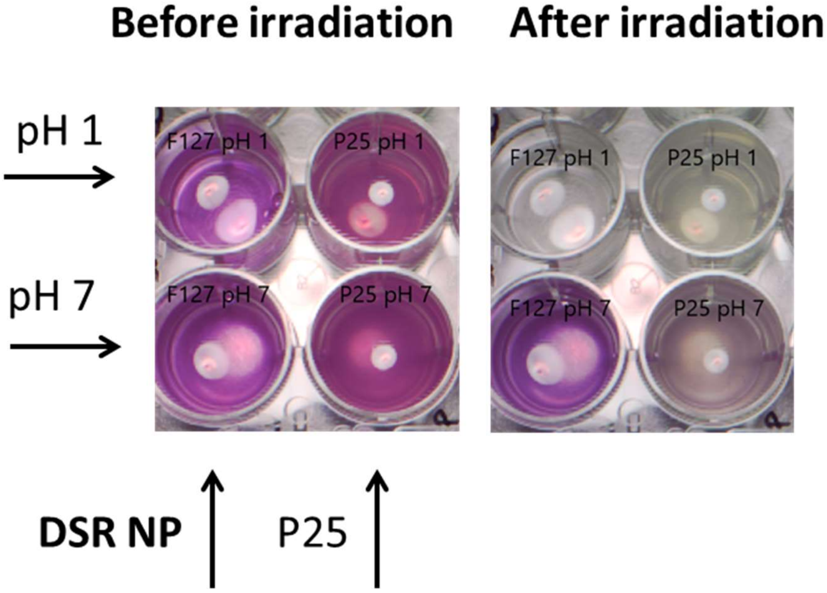

Figure S6*: Photographs of wells containing RhB and either DSR NP or P25 at pH 1 or pH 7 before (left) or after 30 min irradiation (right) with a solar light simulator lamp.*

Detection of hydroxyl radicals via fluorescent probe

In order to understand the initial process and the mechanism of RhB photodegradation, a fluorescent probe (coumarin) for the hydroxyl radical detection was employed. This method is based in the fact that coumarin (COU) is poorly fluorescent, however it readily reacts with hydroxyl radical to produce 7-hydroxycoumarin (7HCOU), a highly fluorescent molecule (see Figure S7). The advantage of using this probe was that coumarin chemical structure carries no charge and 7HCOU emission lies in visible region (emission maximum lies at 460 nm). This method is reported to be rapid, sensitive and specific for hydroxyl radical. [7,8]

For kinetic investigation 6 µL of COU (5 mM) and 42 µL of **DSR NP** (final concentration 0.2 mg/ml) was dissolved in 3 ml of milliQ water. Solution pH was adjusted by HCl or NaOH water solutions. Resulting solution was kept under stirring (900rpm) during measurements. Irradiation (340 nm) is performed using the excitation beam of a conventional spectrofluorometer (HORIBA FluoroMax, intensity $1.1197 \cdot 10^{16}$ photon/s) and the fluorescence of 7HCOU is detected at 460 nm in a L configuration.

Results are reported in figure S8. 7HCOU increasing concentration [7HCOU](t) with respect to the initial one [7HCOU](0), was determined by measuring I_T/I_R, where I_R is emission intensity at 460 nm at time 0 sec, and I_T is emission intensity at 460 nm at irradiation time t sec.

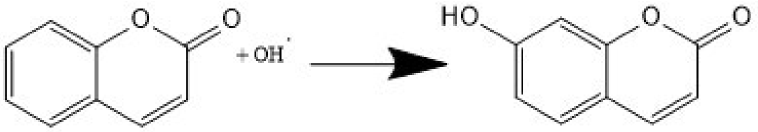

***Figure S7**: Formation of 7-hydroxycoumarin (7HC) by hydroxyl addition to coumarin (COU).*

In order to better compare hydroxyl radical photogeneration, we fitted our data with a pseudo zero-order model according to equation:

$$\frac{I_T}{I_R} = 1 - kt$$

The kinetic rate constants resulting from the fitting are shown in table S1 and they demonstrate that hydroxyl radical photogeneration at pH=3.0 is more than 5 times faster than at pH 6.0.

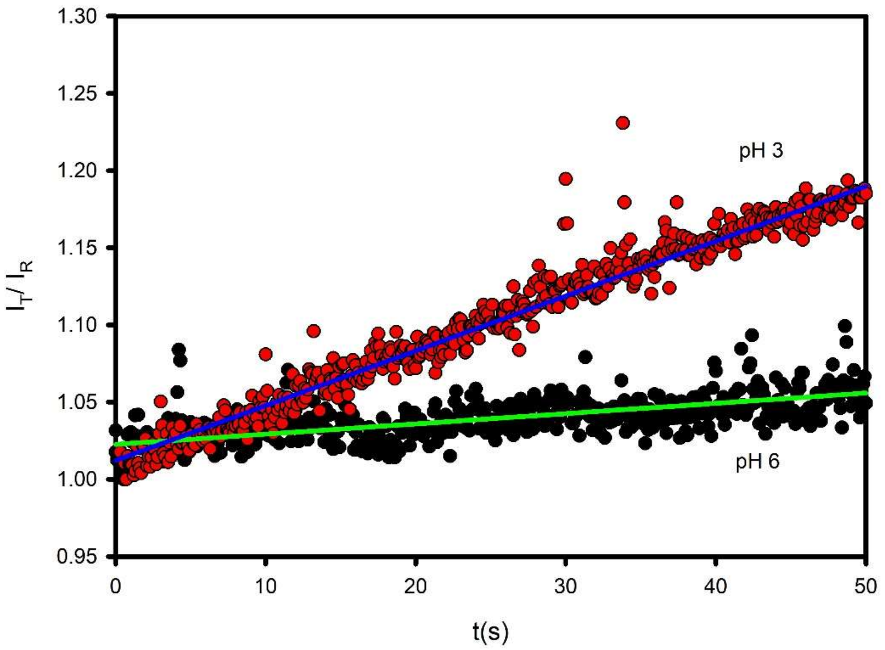

***Figure S8**: Elaboration of hydroxyl radical detection. Comparison of photoactivity at pH 3 and pH 6: signals proportional to the concentration of 7HCOU are plotted as a function of the irradiation time. Hydroxyl radical photogeneration resulted to be five times faster at pH 3 in comparison with pH 6.*

**pH**	***K* $(\cdot 10^{-4})$**
3	7 ± 0.1
6	36 ± 0.3

***Table S1**: Fitted kinetic constant resulted from zero-order model.*

Cell culture

The HeLA human adenocarcinoma cell line was routinely grown in DMEM medium supplemented with 10% heat inactivated fetal bovine serum (FBS), 1% L-glutamine 200 mM and 1% penicillin–streptomycin solution 100 U/mL. Cells were grown at 37 °C in a humidified incubator with 5% CO_2.

Photo-chemical killing of HeLA cells

HeLA cells were seeded in 96 well flat bottom plates and incubated overnight up to 90% confluency. Afterwards, cells were treated with DSR NP, diluted from 1:10 to 1:100 000 in either PBS pH 7 or PBS pH 5, and then irradiated for 10' with light source provided by a natural light bulb (~2 mW/cm^2) or kept under dark conditions. At the end of irradiation, PBS was removed, and cells were incubated in complete medium for 24 h at 37 °C with 5% CO_2. Cell viability was evaluated through MTT assay. All results are expressed as the mean ± standard deviation of at least three independent experiments.

The same experiment was repeated with commercial TiO_2 NP (P25). Results are reported in figure S9 that shows as the cell viability does not change significantly in the investigated concentration interval, upon irradiation, neither at pH 5.0 or 9H 7.0.

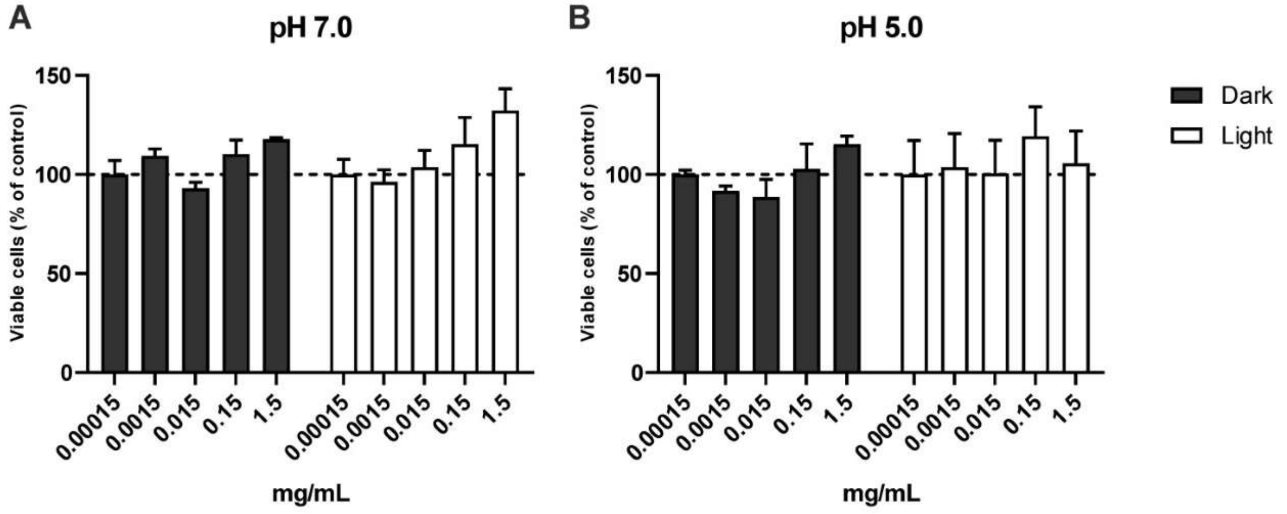

Figure S9 *The killing of cancer cell line by P25 upon photo and chemical stimulation was evaluated on HeLA cell treated with P25 (15 mg/ml) diluted from 1:10 (d10) to 1:100 000 (d100 000) in either A) PBS pH 7 or B) PBS pH 5. After treatment cells were irradiated with white lamp (white bars) or kept under*

*dark conditions (dark grey bars). Cell viability is expressed in percentage with respect to the untreated sample (dashed line). *= p-value < 0.05, ***= p-value < 0.01, ***= p-value < 0.001*

REFERENCES

[1] E. Rampazzo, S. Bonacchi, R. Juris, M. Montalti, D. Genovese, N. Zaccheroni, L. Prodi, D. C. Rambaldi, A. Zattoni, P. Reschiglian, *The Journal of Physical Chemistry B* **2010**, *114*, 14605-14613.

[2] G. Guidetti, D. Giuri, N. Zanna, M. Calvaresi, M. Montalti, C. Tomasini, *ACS Omega* **2018**, *3*, 8122-8128.

[3] M. Montalti, A. Credi, L. Prodi, M. T. Gandolfi, Handbook of Photochemistry (3rd ed.), **2006**, CRC Press.

[4] A. A. Inyinbor, F. A. Adekola and G. A. Olatunji, *S. Afr. J. Chem.*, **2015**, *68*, 115–125.

[5] N. Ghobadi, *International Nano Letters* **2013**, *3*, 2.

[6] M. Qureshi, K. Takanabe, *Chemistry of Materials* **2017**, *29*, 158-167.

[7] Q. Xiang, J. Yu and P. K. Wong, J. Colloid Interface Sci., 2011, 357, 163–167.

[8] A. Gomes, E. Fernandes and J. L. F. C. Lima, J. Biochem. Biophys. Methods, 2005, 65, 45–80.
